# Empathy for pain in individuals with autistic traits during observation of static and dynamic stimuli

**DOI:** 10.3389/fpsyt.2022.1022087

**Published:** 2022-11-16

**Authors:** Yanting Li, Zilong Wei, Min Shao, Mingyu Hong, Di Yang, Longli Luo, Jing Meng

**Affiliations:** ^1^Key Laboratory of Applied Psychology, Chongqing Normal University, Chongqing, China; ^2^Research Center for Brain and Cognitive Science, Chongqing Normal University, Chongqing, China

**Keywords:** autism spectrum disorder, autism, empathy, empathy for pain, event related potential

## Abstract

Previous studies have reported that individuals with autistic traits, like those with Autism Spectrum Disorder (ASD), may have impaired empathic responses when observing static stimuli of others' pain. However, it remains unclear whether individuals with autistic traits exhibit impaired empathy for pain in response to dynamic stimuli. The present study addressed this question by recruiting 529 individuals whose autistic traits were assessed using the autism-spectrum quotient (AQ) questionnaire. Thirty participants who scored within the top 10% and bottom 10% on the AQ were selected into High-AQ and Low-AQ groups, respectively. This study employed painful whole-body action pictures and videos as static and dynamic stimuli. Both groups were instructed to judge whether the models in the stimuli were experiencing pain, and their reaction times, accuracy and event-related potential (ERP) data were recorded. Results showed that the P2 amplitudes were larger in the High-AQ group than in the Low-AQ group when viewing painful static stimuli, while no difference between the two groups was found when viewing painful dynamic stimuli. These results suggest that autistic traits influenced the emotional processing of others' pain in response to static stimuli.

## Introduction

Autism spectrum disorder (ASD) is a neurodevelopmental disorder (1). The condition is persistent and is characterized by repetitive and restrictive patterns in behaviors, activities, or interests in social interactions (2). It has been suggested that ASD exists along a continuum of autistic-like symptoms, such as social-cognitive impairments (3). Autistic traits (symptoms associated with ASD) are distributed in the general population (4). The Autism Spectrum Quotient (AQ) questionnaire is commonly used to assess autistic traits, and higher scores are associated with higher levels of autistic traits (5, 6). Like those with ASD (7), individuals who have autistic traits also show characteristics of reduced sensitivity to social information (8). Due to the similarities between individuals with ASD and those with autistic traits, it may be possible to better understand the social abnormalities observed in the ASD population by studying individuals with autistic traits (4).

Empathy is the ability to understand the feelings and emotions of others (9). Lack of empathy may lead not only to misunderstandings, and apparent insensitivity to others' feelings, but also to fundamental difficulties in social life (10). Empathy consists of two parts: (1) cognitive empathy, which refers to the ability to understand another person's point of view; and (2) emotional empathy, which is the observer's emotional response to another person's mental state (11). The mind-blindness theory of ASD indicates that people with ASD have difficulty understanding the feelings, thoughts, and beliefs of others due to impaired empathy, resulting in atypical social interaction patterns (3). Lack of empathy and difficulty understanding the emotions of others are important criteria for the diagnosis of ASD (12, 13). Some researchers believe that individuals with ASD have cognitive empathy deficits (14), and it has been suggested that impaired implicit endoreceptive reasoning may contribute to those deficits (15). However, some studies have suggested that individuals with ASD exhibit impaired emotional empathy, not cognitive empathy (16, 17). As the inability to feel and show empathy can effectively predict autism and autistic traits (18), research on empathy in individuals with autistic traits can help us better understand autism.

Some studies that have used static stimuli of human expressions have indicated that both individuals with ASD (19, 20), and those with autistic traits (21) may have difficulty experiencing empathy, and hypothesized that this deficit may be the core mechanism underlying their social impairments (22). However, other studies have not found evidence to support this theory in real-life environments (23, 24). When we express emotions with full-body movements and postures, real-life emotions tend to be vivid. It is believed that dynamic displays induce activation of the mirror neuron system, a neuronal network related to empathy, which means that dynamic displays enhance an individual's empathy (25).

Empathy for pain is the ability to perceive and judge the pain of others (26), which includes cognitive and emotional components of understanding others' pain (27). Most previous studies have used static stimuli of others' pain, such as pictures of injured human limbs or faces, to explore empathy for pain in individuals with autistic traits (4, 26, 28–30), because static stimuli of other's pain easily inducing a response of empathy for pain and with high reproducibility (31). Some studies have used dynamic stimuli of others' pain, such as cutting one's hand with a knife while chopping vegetables or getting it caught in a door while closing it to explore empathy for pain (32–34). Usually, both static and dynamic painful stimuli elicit N1, N2, P2, and P3, and late positive potential (LPP) components in ERP studies of empathy for pain. N1, N2, and P2 represent the early emotional component, while P3 and LPP represent the late cognitive component of empathy for pain (35–39). The early involuntary onset of pain empathy for emotion sharing and emotional contagion is reflected in early components, while late pain empathic processing, including pain recognition and judgment of self - others is reflected in late components (40).

Some studies have found that individuals with autistic traits exhibited difficulty recognizing pictures of others' limb pain (30), and produced larger P3 amplitudes when judging pictures of injured human faces (28, 29) compared to controls. This suggests that individuals with autistic traits may devote more cognitive and mental resources to processing others' painful static stimuli, requiring more complex cognitive assessment and control (28, 29). For individuals with ASD or autistic traits, the difficulty in empathy for pain may not be limited to static stimuli of parts of the human body, but may extend to processing information from whole-body postures and actions (41). However, to our knowledge, no study has used dynamic stimuli of whole-body pain to explore empathy for pain in individuals with autistic traits.

As in real-life, pain is dynamic and realistic, we investigated the influence of whole-body painful, dynamic and static stimuli on individuals with autistic traits' empathy for pain. We assumed that if their difficulty in empathy for pain is related to both static and dynamic information, the deficit may relate to their overall empathic ability. However, if the impairment exists only when recognizing painful static, but not dynamic stimuli, the deficit in recognizing others' pain may be related to the decoding of painful static stimuli. It will provide new perspectives regarding empathy in ASD in the future.

## Methods

### Ethics

The study was approved by the Chongqing Normal University Research Ethics Committee, and all procedures were performed in accordance with ethical guidelines and regulations. Written informed consent was provided by all participants prior to participation in the experiment in accordance with the Declaration of Helsinki.

### Participants

A total of 529 adults from the Chongqing Normal University were recruited to complete a paper and pencil version of the Mandarin Version of the AQ questionnaire (5) to identify those with high and low autistic traits. The AQ questionnaire consists of five subscales: social skills, attention shifting, attention to detail, communication, and imagination, each of which is comprised of 10 questions. The questions are answered using a 4-point scale, ranging from “agree completely”, “agree slightly”, “disagree slightly”, to “disagree completely”. Higher scores reflect more autistic traits.

An a priori power analysis using G*Power 3 (42) was conducted using a conservative average of moderate effect sizes from previous sharing empathy for pain studies (Cohen's *d* = 0.79–0.44) (43). The analysis yielded a sample size of *n* = 14 per group to detect a medium effect size of *d* = 0.44 at a standard error probability of α = 0.05 and power of 1–β = 0.95. Following this, two subsets of 30 participants, those exhibiting the top 10% and bottom 10% of AQ scores (26, 44) from the total of 529 adults were randomly selected and divided into High-AQ (*n* = 30, female = 15) and Low-AQ (*n* = 30, female = 15) groups. We invited a total of 63 participants, although three refused. All participants were paid a small sum of money for participating in the experiment. Detailed demographic characteristics of the High-AQ and Low-AQ groups are summarized in Table 1. Criteria for inclusion were: normal or corrected-to-normal vision and no history of neurological or psychiatric disorders.

**Table 1:** Demographic characteristics of the High-AQ and Low AQ groups.

**Group**	**Age (years)**					**AQ Scores**				
	**Min**	**Max**	***M* ±*SD***	***t***	***p***	**Min**	**Max**	***M ±SD***	***t***	***p***
High-AQ	18	26	20.20 ± 2.04	−0.94	0.334	25	33	28.13 ± 1.20	25.47	**< 0.001**
Low-AQ	18	25	20.73 ± 2.20			6	16	14.07 ± 2.27		

AQ, Autism Spectrum Quotient. Statistical results were obtained using independent sample *t*-tests between the High-AQ and Low-AQ groups.

Significant comparisons (*p* < 0.05) are indicated in boldface.

### Stimuli

The stimuli used in the experiment were static and dynamic stimuli whole-body actions showing actors in painful or non-painful situations. Twenty adult actors (10 female), aged between 18 and 24 years (mean ± SD = 22.30 ± 2.12 years) were recruited to record the stimuli. To eliminate distractions, they were asked to dress in a uniform (white T-shirt and black shorts), remove any accessories (e.g., earrings) and to not wear make-up. Before recording, actors received remuneration and voluntarily signed informed consent forms for the use of portrait rights.

#### Dynamic stimuli

Actors were given instructions and guided to express either painful or neutral actions in 1 s durations. Prior to recording, they were encouraged to imagine personal situations to evoke painful and non-painful feelings. During the recording of painful dynamic stimuli, each actor was asked to exhibit painful feelings evoked by imagining pain in different body parts. Similarly, the actors were asked to exhibit neutral feelings in corresponding body parts when recording non-painful dynamic stimuli. Finally, 160 dynamic stimuli were obtained (80 painful and 80 non-painful video clips, recorded by 8 males and 8 females).

Actors were filmed in an evenly lit green-screen studio with an ambient temperature of ~26°C, using a Sony FDR-AXP55 (Sony Group Corporation), at a distance of ~3 m from the actors. The camera height was 1.2 m, and each video clip was 2,160 × 1,280 pixels, using a 60 fps progressive scan.

**Figure 1:**
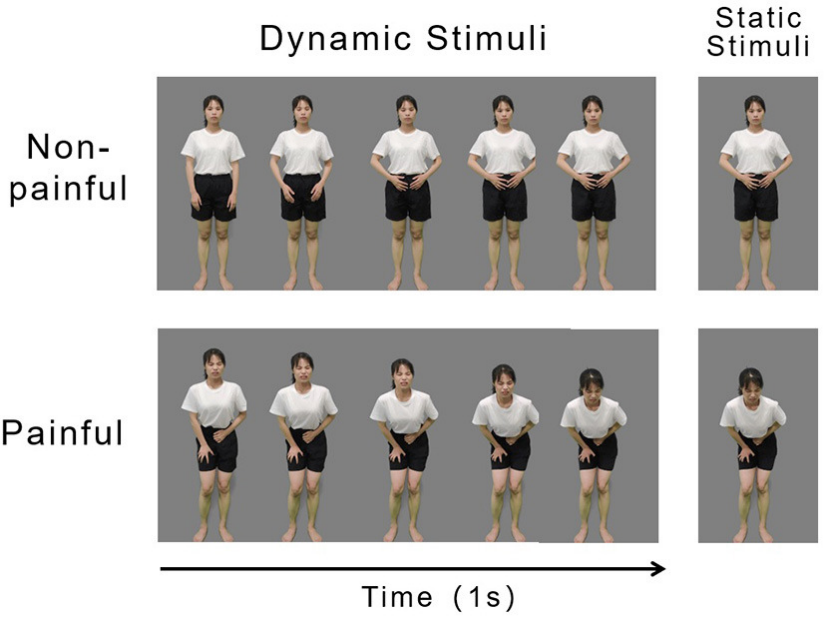
Examples of non-painful **(Top)** and painful **(Bottom)** stimuli. Examples of dynamic **(Left)** and static **(Right)** stimuli.

The video footage was edited using Adobe Premiere Adobe Premiere Pro2020 (Adobe Systems Incorporated). The green background was changed to gray and the actor was isolated from all other contextual information. Each video was edited to 1 s duration, Mp4 format, 768 × 432 pixels, and 60 fps. The luminance, contrast, and color were matched between the painful and non-painful dynamic stimuli. Examples of the dynamic stimuli are displayed in Figure 1, Left panel.

#### Static stimuli

The static stimuli used in the experiment were 160 screenshots that were derived from the 160 dynamic stimuli. A frame of an image that best represented the painful and non-painful feelings of an actor in each dynamic stimulus was cut out and used as static stimuli (examples of which are illustrated in the right panel of Figure 1). Each picture was 15.24 × 27.9 cm (width × height) and 72 pixels per inch.

#### Estimation of the stimuli

To assess how well the static and dynamic stimuli accurately reflected painful and non-painful feelings, seventy adults (35 females and 35 males), aged between 18 and 25 (mean ± SD = 21.96 ± 1.94) years from the Chongqing Normal University were recruited and were asked to evaluate the stimuli. They were not involved in the main experiment. In the experiment we used a 9-point Likert scales to assess: pain intensity (1 = no sensation, 4 = pain threshold, 9 = unbearable pain), emotional valence (1 = very unhappy, 9 = very happy), arousal (1 = extremely peaceful, 9 = extremely exciting), and control (1 = beyond control, 9 = under control). These results are summarized in Table 2.

**Table 2:** The descriptive statistical analysis of dynamic and static stimuli (*M* ±*SD*).

**Dimension**	**Stimuli type**	**Dynamic stimuli**			**Static stimuli**		
		***M ±SD***	***t***	***p***	***M ±SD***	***t***	***p***
Pain intensity	Non-painful	2.24 ± 0.35	81.45	**< 0.001**	2.14 ± 0.34	82.09	**< 0.001**
	Painful	6.31 ± 0.68			6.09 ± 0.65		
Emotional valence	Non-painful	5.01 ± 0.17	−59.64	**< 0.001**	4.89 ± 0.15	−47.05	**< 0.001**
	Painful	3.88 ± 0.25			4.00 ± 0.25		
Arousal	Non-painful	3.11 ± 0.22	57.36	**< 0.001**	2.99 ± 0.24	64.44	**< 0.001**
	Painful	4.84 ± 0.43			4.85 ± 0.40		
Control	Non-painful	7.02 ± 0.18	−48.01	**< 0.001**	6.97 ± 0.17	−51.62	**< 0.001**
	Painful	5.88 ± 0.32			5.71 ± 0.34		

Significant comparisons (*p* < 0.05) are indicated in boldface.

## Procedure

Study participants were seated in a quiet room with an ambient temperature about 26°C. Stimuli were presented on a 24-inch computer screen, using the E-Prime (3.0) program (Psychology Software Tools, Inc, Pittsburgh, PA, USA). All participants were seated about 80 cm from the screen, subtending a visual angle of 5.6° × 10° at viewing.

**Figure 2:**
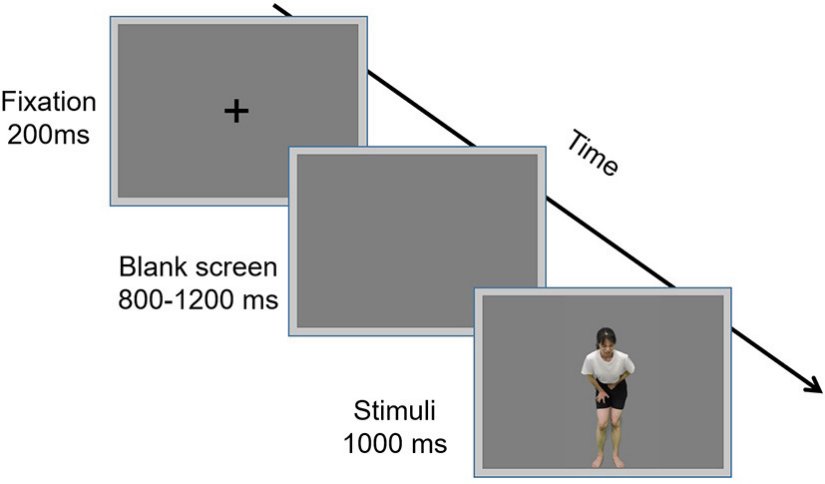
Flowchart describing the experimental design.

Participants were then instructed to assess whether the actor in each stimulus was experiencing pain. At the commencement of each trial, participants were presented with a 800–1,200 ms blank gray screen upon which a 200 ms fixation cross was presented, which was followed by the presentation of a 1,000 ms stimulus. Each stimulus was randomly presented once only. Participants were instructed to respond as accurately and quickly as possible by pressing a key (“1” or “2”) to indicate whether the actor depicted pain. Key-pressing was counterbalanced across participants to control for potential order effects. The inter-trial interval was 1,000 ms. The experiment comprised four blocks with 80 trials per block: two blocks included dynamic stimuli and two blocks included static stimuli. The four blocks were counterbalanced across all participants to control for possible order effects, and there was a 2–5 min break between blocks. The experimental procedure is illustrated in Figure 2.

After the experiment, participants were instructed to rate the attributes of pain intensity (1 = no sensation, 4 = pain threshold, 9 = unbearable pain), and emotional valence (1 = very unhappy, 9 = very happy), for each stimulus, using 9-point Likert scales.

### EEG recording and data analyses

Electroencephalography (EEG) data were recorded from 64 scalp sites using tin electrodes mounted on an actiCHamp system (Brain Vision LLC, Morrisville, NC, US). The electrode on the frontal mastoid was used as a recording reference, and the one on the medial frontal aspect was used as a ground electrode. All electrode impedances remained below 5 kΩ.

EEG data were pre-processed and analyzed *via* MATLAB R2016a (MathWorks, Natick, MA, USA), and the EEGLAB toolbox (45). Continuous EEG signals were band-passed, filtered (0.1–40 Hz), and segmented using a 1,200 ms time window. Time windows of 200 ms before and 1,000 ms after the onset of stimuli were extracted from the continuous EEG. EEG epochs were baseline-corrected by a 200 ms time interval prior to stimuli onset. Epochs with amplitude values exceeding ± 80 μV at any electrode were excluded from the analyses. EEG epochs were also visually inspected, and trials containing significant noise from gross movements were removed–removed epochs accounted for 2.73 ± 4.85% of the total number of epochs. Electro-oculographic artifacts were corrected with an independent component analysis algorithm (46).

After confirming scalp topographies in both the single-participant and group-level event-related potential (ERP) waveforms, as well as on the basis of previous studies (47, 48), dominant ERP components and the electrodes included in the statistical analyses were identified as follows: N1, N2, and P2 (F1, Fz, F2, FC1, FCz, and FC2); P3 and late positive peak (LPP) (P3, Pz, P4, PO3, POz, and PO4). Amplitudes of N1, N2, P2, and P3 components were calculated as mean amplitudes with a latency interval of peak ± 20 ms at electrodes displaying maximal responses. The LPP was extracted within a time window of 400–600 ms.

Statistical analyses

Data analyses were performed using SPSS 15.0 software (IBM Corp., Armonk, NY, USA). Behavioral data [reaction times (RTs), accuracies (ACC)], subjective ratings (pain intensity and emotional valence) and ERP data (amplitudes of dominant ERP components) were analyzed using a three-way repeated-measure ANOVA. The within-participants' factors included: “Stimuli type” (dynamic stimuli, static stimuli) and “Pain” (painful, non-painful), with the between-participants' factor being “Group” (High-AQ, Low-AQ). The degrees of freedom for the F-ratios were corrected according to the Greenhouse-Geisser method. If the interactions between the three factors were significant, simple effects between groups analyses were performed for each condition. In total, nine 3-way ANOVAs were calculated.

Finally, we investigated the relationship between neural responses and autistic traits by assessing the correlation between participants' behavioral data and ERP amplitudes (N1, P2, N2, P3, and LPP), using Pearson's Correlation. To account for multiple comparisons, the false discovery rate (FDR) procedure (49) was used to correct *p* value.

## Results

### Behavioral results

#### Reaction time

RTs were modulated by the main effects of “Stimuli type” (*F*_(1, 58)_ = 11.87, *p* = 0.001, $\eta_{\text{p}}^{2}$ = 0.17) and “Pain” (*F*_(1, 58)_ = 29.92, *p* < 0.001, $\eta_{\text{p}}^{2}$ = 0.34). RTs were longer for dynamic stimuli (1,156.12 ± 55.43 ms) than for static stimuli (1,016.18 ± 44.81 ms), while RTs were longer for non-painful stimuli (1,185 ± 55.74 ms) than for painful stimuli (986.45 ± 42.59 ms).

#### Accuracies

ACCs were modulated by the main effect “Pain” (*F*_(1, 58)_ = 11.37, *p* = 0.001, $\eta_{\text{p}}^{2}$ = 0.16) with the ACCs for painful stimuli (91.40 ± 1.60 %) being higher than those for non-painful stimuli (88.00 ± 1.80 %).

#### Pain intensity

Pain intensity ratings were modulated by the main effect of “Pain” (*F*_(1, 58)_ = 1,028.93, *p* < 0.001, $\eta_{\text{p}}^{2}$ = 0.95). The pain intensity for painful stimuli (6.35 ± 0.12) was higher than that of the non-painful stimuli (2.05 ± 0.08).

#### Emotional valence

Emotional valence ratings were modulated by the main effects of “Stimuli type” (*F*_(1, 58)_ = 6.73, *p* = 0.012, $\eta_{\text{p}}^{2}$ = 0.10), and “Pain” (*F*_(1, 58)_ = 58.66, *p* < 0.001, $\eta_{\text{p}}^{2}$ = 0.50). The emotional valence was more negative for static stimuli (4.28 ± 0.11) than for dynamic stimuli (4.37 ± 0.10), while the emotional valence was more negative for painful stimuli (3.60 ± 0.15) than for non-painful stimuli (5.06 ± 0.14).

### ERP results

**Figure 3:**
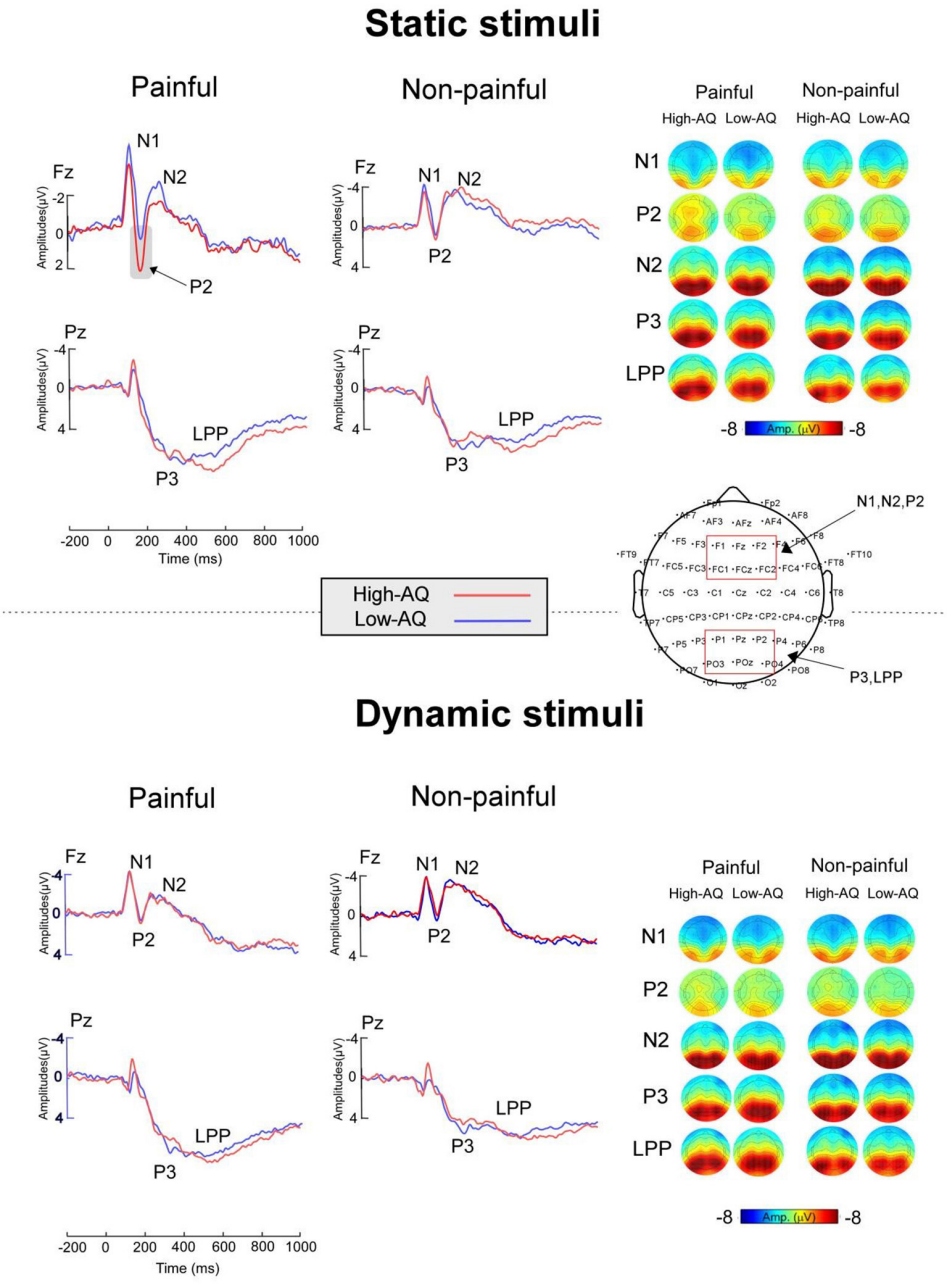
ERP waveforms **(Right)** and scalp topography distributions **(Left)** exhibited by the High-AQ (blue lines) and Low-AQ (red lines) groups in response to static stimuli **(Top)** and dynamic stimuli **(Bottom)**. Electrodes used to estimate the ERP amplitudes are marked using the red squares on their respective topographic distributions.

**Figure 4:**
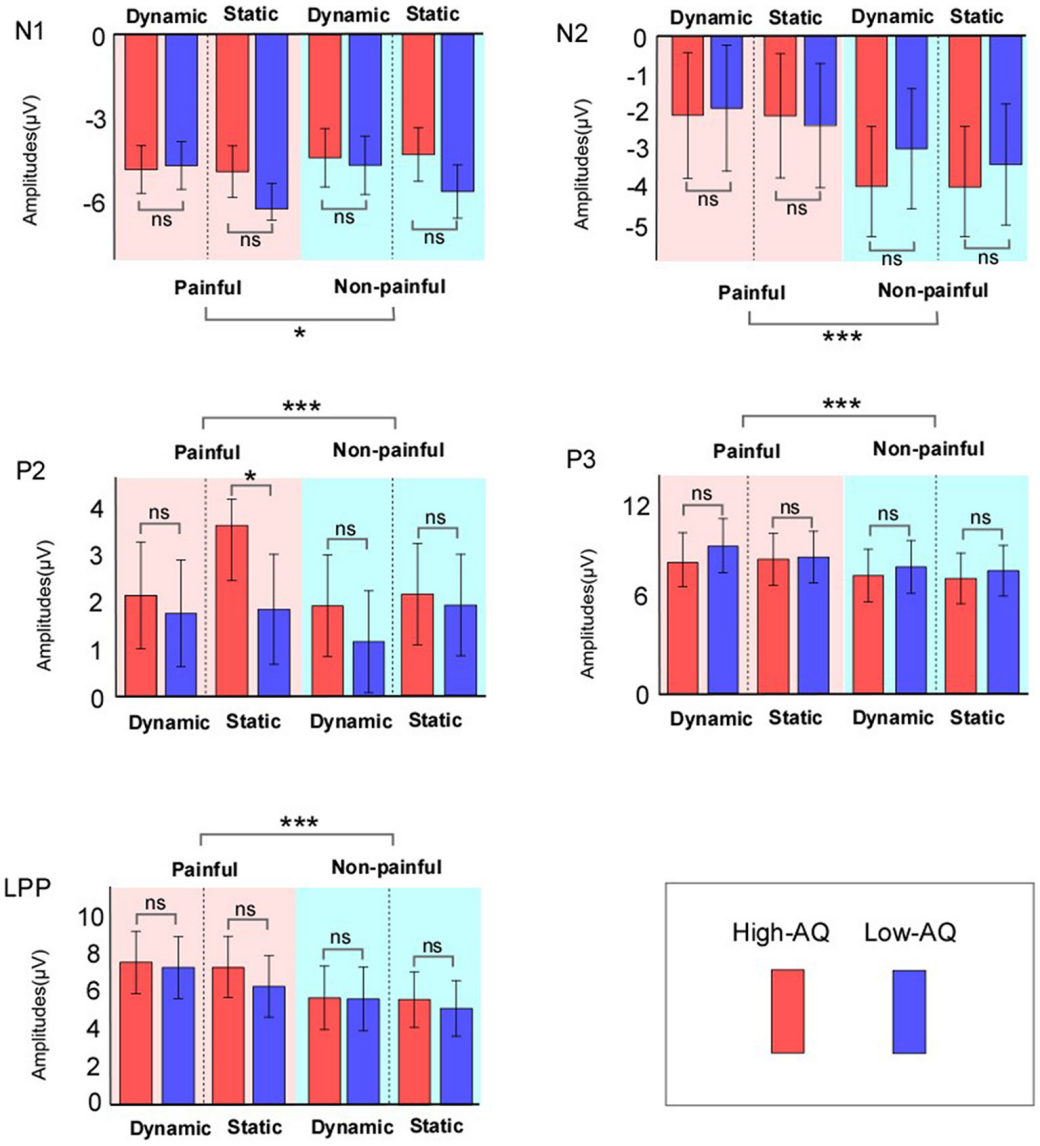
ERP amplitudes of High-AQ (blue bar) and Low-AQ (red bar) groups in response to static and dynamic stimuli. Data in the charts are expressed as Mean ± 2SEM. ns: *p* > 0.05, *p < 0.05, **p < 0.01, ***p < 0.001.

ERP waveforms and scalp topographies are shown in Figure 3, and bar charts for ERP amplitudes are shown in Figure 4. Results of the statistical analyses of the ERP amplitudes are summarized in Table 3.

**Table 3:** Summary of statistical analyses of ERP amplitudes.

	**N1**			**N2**			**P2**			**P3**			**LPP**		
	***F***	***p***	** $\eta_{\text{p}}^{2}$ **	***F***	***p***	** $\eta_{\text{p}}^{2}$ **	***F***	***p***	** $\eta_{\text{p}}^{2}$ **	***F***	***p***	** $\eta_{\text{p}}^{2}$ **	***F***	***p***	** $\eta_{\text{p}}^{2}$ **
Group	1.33	0.254	0.02	0.13	0.723	< 0.01	1.25	0.268	0.02	0.24	0.624	< 0.01	0.20	0.660	< 0.01
Stimuli type	2.48	0.121	0.04	0.41	0.527	0.01	5.13	**0.027**	0.08	0.57	0.452	0.01	1.10	0.299	0.02
Pain	5.38	**0.024**	0.09	38.70	**< 0.001**	0.40	11.61	**0.001**	0.17	34.44	**< 0.001**	0.37	46.49	**< 0.001**	0.45
Group × Stimuli type	7.41	**0.009**	0.11	0.40	0.532	0.01	0.60	0.443	0.01	0.55	0.460	0.01	0.67	0.415	0.01
Group × Pain	0.34	0.564	0.01	3.09	0.084	0.05	2.92	0.093	0.05	0.02	0.899	< 0.01	0.71	0.404	0.01
Stimuli type × Pain	1.97	0.166	0.03	< 0.01	0.971	< 0.01	1.06	0.307	0.02	< 0.01	0.986	< 0.01	1.53	0.222	0.03
Group × Stimuli type × Pain	0.67	0.416	0.01	0.01	0.934	< 0.01	12.19	**0.001**	0.17	1.59	0.212	0.03	0.40	0.532	0.01

Notes: Results were obtained using repeated-measures ANOVA with the within-participant factors of “Stimuli type” (dynamic stimuli, static stimuli) and “Pain” (painful, non-painful), as well as the between-participants factor of “Group” (High-AQ, Low-AQ).

Significant comparisons (*p* < 0.05) are indicated in boldface.

N1 amplitudes were modulated by “Pain” (*F*_(1, 58)_ = 5.38, *p* = 0.024, $\eta_{\text{p}}^{2}$ = 0.09), and painful stimuli (−5.03 ± 0.27 μV) elicited larger N1 amplitudes than non-painful stimuli (−4.63 ± 0.33 μV). N1 amplitudes were also modulated by the interaction between “Group” and “Stimuli type” (*F*_(1, 58)_ = 7.41, *p* = 0.009, $\eta_{\text{p}}^{2}$ = 0.11), with static stimuli (−5.64 ± 0.42 μV) eliciting larger N1 amplitudes than dynamic stimuli (−4.69 ± 0.45 μV) in the Low-AQ group. No other main effects or interactions were found (*p* > 0.05).

N2 amplitudes were modulated by “Pain” (*F*_(1, 58)_ = 38.70, *p* < 0.001, $\eta_{\text{p}}^{2}$ = 0.40), with non-painful stimuli (−3.56 ± 0.53 μV) eliciting larger N2 amplitudes than painful stimuli (−2.09 ± 0.55 μV).

P2 amplitudes were modulated by the main effects of “Stimuli type” (*F*_(1, 58)_ = 5.13, *p* = 0.027, $\eta_{\text{p}}^{2}$ = 0.08) and “Pain” (*F*_(1, 58)_ = 11.61, *p* = 0.001, $\eta_{\text{p}}^{2}$ = 0.17). Static stimuli (2.40 ± 0.38 μV) elicited larger P2 amplitudes than dynamic stimuli (1.77 ± 0.38 μV), and painful stimuli (2.37 ± 0.37 μV) elicited larger P2 amplitudes than non-painful stimuli (1.79 ± 0.36 μV). P2 amplitudes were also modulated by the interaction between “Group”, “Stimuli type”, and “Pain” (*F*_(1, 58)_ = 12.19, *p* = 0.001, $\eta_{\text{p}}^{2}$ = 0.17). Simple effect analyses indicated that P2 amplitudes were larger in the High-AQ group (3.65 ± 0.59 μV) than in the Low-AQ group (1.87 ± 0.59 μV, *p* = 0.035) in response to painful static stimuli. However, no group difference was found in response to other stimuli (i.e., non-painful static stimuli, painful and non-painful dynamic stimuli) (*p* > 0.05).

P3 and LPP amplitudes were modulated by “Pain” (P3: *F*_(1, 58)_ = 34.44, *p* < 0.001, $\eta_{\text{p}}^{2}$ = 0.37; LPP: *F*_(1, 58)_ = 46.49, *p* < 0.001, $\eta_{\text{p}}^{2}$ = 0.45), with painful stimuli (P3: 8.65 ± 0.56 μV; LPP: 7.21 ± 0.55 μV) eliciting larger amplitudes than non-painful stimuli (P3: 7.58 ± 0.55 μV; LPP: 5.59 ± 0.54 μV).

### Correlation between ERP data and behavioral data

Results of the correlation analysis indicated that participant's AQ scores were positively correlated with the P2 amplitudes of painful static stimuli (*r* = 0.28, *p* = 0.033). A significant correlation between P2 amplitudes and RTs for painful dynamic stimuli (*r* = −0.38, *p* = 0.011) was found. No other significant correlations were found between ERP data and behavioral data (*p* > 0.05). The correlation results for ERP amplitudes and behavioral data (painful dynamic/static stimuli) and AQ scores are presented in Table 4.

**Table 4:** Correlation between ERP amplitudes and behavioral data.

**Stimuli type**	**ERP components**	**AQ**	**RTs**	**ACC**	**Pain intensity**	**Emotional valence**
Dynamic	N1	−0.07	−0.10	−0.02	−0.01	−0.08
	P2	< 0.01	−0.33*	−0.08	−0.03	0.10
	N2	0.04	−0.08	−0.17	−0.11	0.01
	P3	−0.12	−0.02	0.02	−0.01	0.02
	LPP	< 0.01	−0.11	0.03	< 0.01	−0.05
Static	N1	0.24	−0.11	0.11	−0.05	0.13
	P2	0.07	−0.25	0.07	−0.05	0.13
	N2	0.28*	0.05	0.03	−0.04	0.01
	P3	−0.04	−0.05	0.13	0.06	0.03
	LPP	0.09	−0.08	0.12	0.07	0.03

^*^p < 0.05.

## Discussion

In order to explore the potential influence of autistic traits on empathy for pain when observing dynamic and static stimuli, we investigated the behavioral and neural responses to others' pain in individuals with autistic traits utilizing videos and pictures of painful whole-body actions as experimental materials. Results showed that P2 amplitudes were larger in the High-AQ group than in the Low-AQ group when viewing painful static stimuli only and no difference between the two groups was found in response to dynamic stimuli. These results suggest that the empathic difficulties experienced by individuals with autistic traits may be influenced by the type of stimuli. In addition, the altered decoding process when interpreting painful static stimuli may be an important cause of difficulties individuals with autistic traits have when identifying others' pain in experimental settings.

Consistent with previous studies (35–39), the present study found that both behavioral (RTs, ACCs) and ERP (N1, N2, P2, P3, and LPP amplitudes) results were significantly modulated by the main effect of “Pain” as participants responded more quickly and more accurately, and showed larger ERP amplitudes to painful than to non-painful stimuli. In previous studies of empathy for pain, a subset of fixed ERP components have been shown to be good indicators. Examples include the N1, P2, and N2 components, which arise above the frontoparietal lobe and reflect early pain perception and emotion sharing, and the P3 and LPP components, which arise above the parieto-occipital lobe and reflect later cognitive appraisal (50). Like previous studies which used painful pictures or videos of human limbs (38, 51, 52), faces (53), and robot hands (52), the present study utilized dynamic and static stimuli of human whole-body actions to demonstrate feelings of pain and elicit similar empathic responses to others' pain.

In previous studies, individuals with high scores on the AQ questionnaire were considered to have autistic traits (26, 28, 29, 54, 55) and it was used to select participants in the present study. The P2 amplitudes in the High-AQ group were larger than those in the Low-AQ group when they viewed painful static stimuli, while the P2 amplitudes of the two groups did not differ in response to painful dynamic stimuli. In addition, P2 amplitudes in response to painful static stimuli were positively correlated with participants' AQ scores, with higher AQ scores corresponding to larger P2 amplitudes in response to painful static stimuli. As P2 represents the emotional component of empathy for pain (56, 57) our findings suggest that emotional processing in response to painful static stimuli may be influenced by autistic traits, and individuals with autistic traits require more mental resources for emotional processing when viewing painful static stimuli or vice versa.

However, no differences were found between the groups in response to painful dynamic stimuli in the present study, which may be because the dynamic stimuli provided more real-world information than the static stimuli. Previous studies have also found individuals with ASD did not exhibit empathic deficits in real-world environments (58), but exhibited empathic difficulties in response to static stimuli in an experimental environment (59). This evidence suggests that the empathy deficit seen in people with ASD and autistic traits may be related to the decoding of emotional painful static stimuli. Previous research has shown that an important feature of mirror neurons is the link between vision and movement. When an individual sees an action performed by another person, the neurons representing that action are activated in the individual's premotor cortex. Thus, individuals are better able to understand the actions performed by others (60). Dynamic simulation of pain provides more detail than static simulation, such as a change in one's facial? expression when feeling pain, and rubbing the painful area when feeling pain. Empathy impairment in individuals with ASD may be related to the level of mirror neural system involvement, and to the type of stimuli used (61). For example, a previous study has found that activation of the mirror neural system was associated with emotional empathy (62), and lower levels of mirror neural system involvement in individuals with ASD may result in greater difficulty decoding painful static stimuli when processing such stimuli. The observation that individuals with ASD do not exhibit empathic deficits when processing painful dynamic stimuli may account for the better decoding of dynamic pain stimuli that was found in individuals with autistic traits in the present study.

Finally, while our study was methodologically sound and conducted in a rigorous manner, there are several limitations that should be acknowledged. First since we focused on the difference in temporal resolution between dynamic and static stimuli in this study, we chose EEG rather than fMRI. The ERP technique used in this study did not provide evidence regarding the area of brain activation, which may be further explored in future studies using fMRI. Second, the participants in the present study were individuals with autistic traits, and future investigations may benefit by including individuals with ASD. Finally, it has also been suggested that individuals with ASD have difficulty empathizing because of alexithymia, rather than an impairment in empathy. Alexithymia has a relatively high prevalence rate in the ASD population, and is characterized by individuals' difficulty identifying and describing their emotional states (12, 13). However, this issue was not analyzed in our experiments, and we plan to investigate it in future studies.

## Conclusions

This study investigated the influence of autistic traits on empathy for pain when viewing dynamic and static stimuli. Our findings of larger P2 amplitudes by the High-AQ group compared to the Low-AQ group in response to painful static, but not dynamic stimuli, suggests that individuals with autistic traits may have altered emotional processing in response to others' painful static stimuli.

## Data availability statement

The original contributions presented in the study are included in the article/supplementary materials, further inquiries can be directed to the corresponding author.

## Ethics statement

The studies involving human participants were reviewed and approved by Chongqing Normal University Research Ethics Committee. The patients/participants provided their written informed consent to participate in this study. Written informed consent was obtained from the individual(s) for the publication of any potentially identifiable images or data included in this article.

## Author contributions

YL: conceptualization, methodology, software, data curation, and writing-original draft preparation. ZW and MS: editor of the revised manuscript. MH: writing-original draft preparation. DY: methodology, software, and writing-original draft preparation. LL: software and data curation. JM: conceptualization, methodology, funding acquisition, and writing-reviewing and editing. All authors contributed to the article and approved the submitted version.

## Funding

This work was supported by the Ministry of Education in China, Humanity and Social Science Youth Foundation Project (Grant number 19YJC190016) and Fundamental Research Funds for the National Natural Science Foundation of China (31400882).

## Conflict of interest

The authors declare that the research was conducted in the absence of any commercial or financial relationships that could be construed as a potential conflict of interest.

## Publisher's note

All claims expressed in this article are solely those of the authors and do not necessarily represent those of their affiliated organizations, or those of the publisher, the editors and the reviewers. Any product that may be evaluated in this article, or claim that may be made by its manufacturer, is not guaranteed or endorsed by the publisher.

## Abbreviations

ASD: Autism spectrum disorder
AQ: Autism Spectrum Quotient
ERP: Event-related Potentials
DSM-5: Diagnostic and Statistical Manual of Mental Disorders (5th ed.)
IAPS: International Affective Picture System
CAPS: Chinese Affective Picture System
ISI: inter-stimulus interval
ANOVA: Analysis of variance
EEG: Electroencephalography
ACCs: Accuracies
RTs: Reaction times.
